# Lifetime climate impacts of diet transitions: a novel climate change accounting perspective

**DOI:** 10.3390/su13105568

**Published:** 2021-05-17

**Authors:** Jonathan E. Barnsley, Chanjief Chandrakumar, Carlos Gonzalez-Fischer, Paul E. Eme, Bridget E. P. Bourke, Nick W. Smith, Lakshmi A. Dave, Warren C. McNabb, Harry Clark, David J. Frame, John Lynch, John R. Roche

**Affiliations:** 1 Ministry for Primary Industries, PO Box 2526, Wellington 6140, New Zealand; 2 New Zealand Agricultural Greenhouse Gas Research Centre, Private Bag 11008, Palmerston North 4442, New Zealand; 3 Riddet Institute, Massey University, Private Bag 11 222, Palmerston North 4442, New Zealand; 4 Victoria University of Wellington, PO Box 600, Wellington 6140, New Zealand; 5 Department of Physics, University of Oxford, Oxford, OX1 3PU, United Kingdom; 6 Department of Biological Sciences, University of Auckland, Auckland 1010, New Zealand

**Keywords:** dietary change, greenhouse gas emissions, climate change, GWP*, New Zealand

## Abstract

Dietary transitions, such as eliminating meat consumption, have been proposed as one way to reduce the climate impact of the global and regional food systems. However, it should be ensured that replacement diets are indeed nutritious and that climate benefits are accurately accounted for. This study uses New Zealand food consumption as a case study for exploring the cumulative climate impact of adopting the national dietary guidelines and the substitution of meat from hypothetical diets. The new GWP* metric is used as it was designed to better reflect the climate impacts of the release of methane than the *de facto* standard 100-year Global Warming Potential metric (GWP100). A transition at age 25 to the hypothetical dietary guideline diet reduces cumulative warming associated with diet by 7 to 9% at the 100^th^ year compared with consuming the average New Zealand diet. The reduction in diet-related cumulative warming from the transition to a hypothetical meat-substituted diet varied between 12 and 15%. This is equivalent to reducing an average individual’s lifetime warming contribution by 2 to 4%. General improvements are achieved for nutrient intakes by adopting the dietary guidelines compared with the average New Zealand diet; however, the substitution of meat items results in characteristic nutrient differences, and these differences must be considered alongside changes in emission profiles.

## 1 Introduction

Producing food for growing populations with changing dietary patterns presents one of the greatest global challenges of this century [1–3]. This challenge is interconnected with a need to manage our environments and ecosystems sustainably. It is estimated that agriculture occupies around 40% of global ice-free land [4], and that food production results in 21 to 37% of global greenhouse gas (GHG) emissions annually [5] (as commonly aggregated using GWP100), and around 70% of global water withdrawals [6]. Of environmental issues related to the global production of food, anthropogenic climate change is arguably most pressing. A recent study by Clark, *et al.* [7] has reported that, in addition to the complete cessation or cancellation of fossil fuel emissions from all sources, achieving the 1.5 and 2°C climate change targets would necessitate mitigation of food system emissions.

There is ongoing research exploring potential solutions to the nutritional and sustainability issues that challenge the global food system. A substantial field of research has focused on consumption aspects, particularly in developed regions and, generally, replacing animal-source foods with plant-source alternatives as one way to reduce the food systems contribution to climate change [8–15], alongside other environmental impacts. However, important caveats with respect to nutrition and other factors of food systems have been acknowledged [16–20]. For example, the Payne, Scarborough and Cobiac [16] study highlights that diets with lower GHG emissions can be highly heterogeneous with respect to nutrients, micronutrients and health outcomes, and that undesirable outcomes for sugar and micronutrient intakes can be common. For this reason, recommended shifts towards low GHG diets should be augmented with dietary advice to ensure micronutrient requirements are met either through supplementation and or combination of different plant-sourced foods [12,21,22].

A key methodological limitation quantifying climate change impacts is in the accurate representation of atmospheric warming through the, so-called, GHG emission ‘metrics’; this contention has been the subject of ongoing debate [23–26]. Currently, the GWP100 metric [27] is used as a *de facto* standard in literature and explores the radiative contribution associated with the production life cycle of different food items [28,29] and more broadly [30,31]. While GWP can represent a straightforward indication of radiative contribution of an emission pulse of a particular gas over a certain period, when compared with carbon dioxide (CO_2_), it does not clearly reveal the cumulative impacts of ongoing emissions of short-lived GHGs, such as methane (CH_4_) [32–34]. This is problematic, given the large CH_4_ emission component for some food items, particularly ruminant animal-sourced foods and rice [15]. A new metric called GWP* has been designed to address some of these issues by primarily considering the flow dynamics of CH_4_, given its relatively short lifetime in the atmosphere, in addition to stock impacts related to slow climatic response [32–34]. There has been a growing interest to explore climate impacts using the GWP* metric and recently, Ridoutt [35] has applied GWP* to life cycle assessments of food items and by Ridoutt, *et al.* [36] to dietary emissions estimates.

Although global studies can provide general guidelines, it is important to explore region-specific nuances, as local food systems and societies have particular characteristics to appreciate. For example, New Zealand makes for an interesting case study to test global observations, as it is generally thought to have more efficient and less GHG emission intensive food production [37], with animal-sourced foods being a significant proportion of total production [38] and a large proportion of GHG emissions being CH_4_ [39]. The New Zealand government provides official dietary guidelines [40], and recently the climate impact of a range of hypothetical diet scenarios that conform to the New Zealand dietary guidelines were explored by Drew, Cleghorn, Macmillan and Mizdrak [13]. This study reported a positive relationship between the inclusion of animal-source foods in hypothetical diets and dietary GHG emissions using the GWP100 metric, and the scenarios explored were estimated to provide 4 to 42% emission reductions compared with the average New Zealand scenario. Drew, Cleghorn, Macmillan and Mizdrak [13] highlight the non-prescriptive nature of the dietary guidelines allows for a plethora of different scenarios that generally convey improved public health outcomes compared with the average New Zealand diet, variation in terms of their daily emission intensities; however, differences in atmospheric warming between short- and long-lived GHGs were not considered and expected variance in nutrient profiles was not explored.

In this study we explore the mitigation potential of dietary change in New Zealand, by analysing the cumulative atmospheric warming impact of one individual adopting the national dietary guidelines and the substitution of meat. The nutrient benefits and trade-offs of the dietary transition are also explored. This work uses the GWP* metric to explore how the warming impact of CH_4_ contributes to dietary climate impact, and contrasts this with the *de facto* standard GWP100. To the best knowledge of the authors, this represents the first study to use GWP* to explore the cumulative climate impact of dietary transitions. This work explores caveats for how different diets are compared (i.e., energy or nutrient basis) and what happens when hypothetical diets have significantly different GHG compositions (i.e., varying proportions of CH_4_ using meat types). Context is provided by examining how the atmospheric warming impacts of dietary change compared with an individual’s wider consumption profile, how an individual’s dietary profile is related to a larger collective (i.e., as part of a family).

## 2 Methods

Full methodological details for this study is provided in the appendix. In summary, the diet framework used in this work is based on the New Zealand Total Diet Study [41,42], and the average simulated adult diet (denoted ‘Current’) was modified based on serving sizes of food groups (i.e., discretionary items, dairy, fruit, grains, vegetables and non-discretionary meat) to give an improved baseline diet that follows the 2015 New Zealand Eating and Activity Guidelines [40] (denoted ‘DG’ for ‘dietary guidelines’). A hypothetical meat substituted diet (denoted ‘DG -Meat’) was generated by taking the DG diet and reducing daily consumption for all meat-based foods to zero, followed by increases in legume, dairy, nut and egg foods using a ratio of 2:1:1:1, respectively, to replace the bioavailable protein deficit resulting from the removal of meat-based foods. To allow the DG and DG -Meat diet to be directly comparable to the Current reference diet, a compensation method is required. This typically involves adjusting the scenarios to be equal in energy [12–14] (2211 kcal per day, in this study) and alternatively the diets can be made equal in bioavailable protein (83.0 g per day, in this study); both are explored. The impact of specific types of meat was also explored, as a sensitivity analysis by exchanging (using bioavailable protein compensation) all meat-based foods in the hypothetical DG diet with only foods of target specific type (e.g., beef and pork). Nutrient data were generated using the New Zealand FOODFiles database [43,44], which is a comprehensive collection of nutrient data for New Zealand foods. To achieve this, the food items of the diet framework were matched to up to three entries of the FOODFiles database and the associated range of nutrient values. As a cross-validation, comparisons were made between the nutrient data generated in this work and several macro- and micro-nutrient values generated for the Total Diet Study (Table A1). For the average adult, average teenager, child, toddler and infant life stages, a generally good agreement was evident (~±5%). For both total fats and saturated fats, the nutrient data generated in this work was ~10% lower, indicating some discrepancies between the two accounting approaches for this nutrient type. To account for the differences in the bioavailability of protein associated with different types of food items, digestible indispensable amino acid score (DIAAS) scaling factors [45] were applied to give a more representative measure of the value of protein from a given food. The protein value, including bioavailability corrections, was used when generating diets.

Life cycle assessment (LCA) data used in this study are based on the database accumulated by [13], with some modifications. Yearly diet-related emissions for a given diet scenario were calculated by matching each common food for a given food category. To assign a proportion of CH_4_ from the production and processing stages to so-called ‘methane-intensive’ foods, data from the original reference as listed in Drew, Cleghorn, Macmillan and Mizdrak [13], or as provided by the authors was used. In some cases, a more recent study was available, and this was used instead (Table ESI6). The values used for ‘methane-intensive’ foods were recalculated from the original literature values using Intergovernmental Panel on Climate Change (IPCC) Assessment Report 5 GWP100 values without climate-carbon feedbacks [30]. To convert between retail and edible weights, the edible portion was provided by the FOODFiles database, the preparation data were either sourced from the Total Diet Study, FOODFiles or using a reference product. The weight change due to cooking was primarily from FOODFiles and supplemented with Australian [46] and US [47] data from similar databases. The [15] global LCA database was used to gap fill for the CH_4_ proportion which were not accounted for by the ‘methane-intensive’ foods from the Drew et al. 2020 database, or when the original literature was not accessible, as is the case for rice. Where applicable, this database also allowed use of the IPCC Assessment Report 5 GWP100, without carbon-climate feedback equivalence values [30] for consistency. Several composite or dish foods in the Total Diet Study item list had no relevant matches (e.g., hamburger or sushi) so a mixture of primary foods was used to generate these specific food entries (Table ESI2). To account for food wastage (i.e., post-retail), product-level waste factors from the UK-based Waste and Resources Action Programme study [48] were used. Detailed information on all the diets is presented in Tables ESI3, ESI4 and ESI5.

New Zealand population data including average life expectancy at birth of 82 years [49] were used to model the individual average lifetime trajectory. Diets were scaled by energy or protein over the different life stages to substantially reduce modelling complexities, based on the correlation of GHG emissions and energy or protein consumption seen for the Total Diet Study typical diets (Figure A2). This scaling approach was achieved based on the energy or protein consumption from the New Zealand Adult and Children’s Nutrition Surveys [50,51] with backfilling for the ages 0-5 based on the Australian and New Zealand dietary nutrient reference values [52]. The transition from the Current diet to the hypothetical diets was modelled to take place at year 25 because this is a life stage where an individual is likely to have dietary choice (i.e., likely to be responsible for their own diet) and so that the transition occurred at a relatively early stage in the lifetime, but after critical development periods. Given that a lifetime is finite, dynamics for the individual scenario will vary compared with an indefinite emission series at collective-levels (e.g., for populations). To bridge the findings of the individual scenario, a longer multigenerational family scenario is also explored as a sensitivity analysis. For this scenario, the New Zealand average fertility values were used including the total fertility rate (1.75 births per woman) and the median age of women having a baby of 31 years [49]. Emission responsibility (Equation A1) is assumed to be completely associated to the 0^th^ generation’s direct family line and for each successive generation after the 1^st^ generation, the generational responsibility changes due to the requirement of partners to generate offspring (Table A2).

Annual emissions were calculated based on the consumption of a given diet for short- (CH_4_) and long-lived GHG categories (CO_2_, and nitrous oxide, N_2_O). Using these values, the cumulative climate impact was estimated using the GWP100 metric for long-lived GHGs and GWP* metric for the short-lived CH_4_. These metrics used the IPCC 2013 equivalence values without climate-carbon feedbacks, which correspond to 28 for CH_4_ and 265 for N_2_O. The GWP* metric is used with the recommended values [33] for the flow term, stock term and the emission rate timeframe, as these terms were reported to give the best approximation of CH_4_ temperature response over Representative Concentration Pathways 2.6 to 6. The GWP* equation calculates CO_2_ warming equivalents (CO_2we_) and is used here is as follows:

(Equation 1) $$E_{CO_{2}we}=\left[\underset{`\text{Flow}`}{r\times \frac{\text{Δ}E_{CH_{4}}}{\text{Δ}t}\times H}+\underset{`\text{Stock}`}{s\times E_{CH_{4}}}\right]\times GWP_{100}$$

where *r* equals the weighting for flow rate (0.75), *ΔECH_4_* equals the difference in emissions of CH_4_ over the timescale of flow, *t* equals the timescale of flow in years (20), *H* is the time horizon corresponding to the GWP used (in this case100-years), *s* equals the weighting for stock (0.25), *E_CH_4__* is the emissions of CH_4_, and *GWP100* is the 100-year Global Warming Potential of CH_4_.

**Figure 1:**
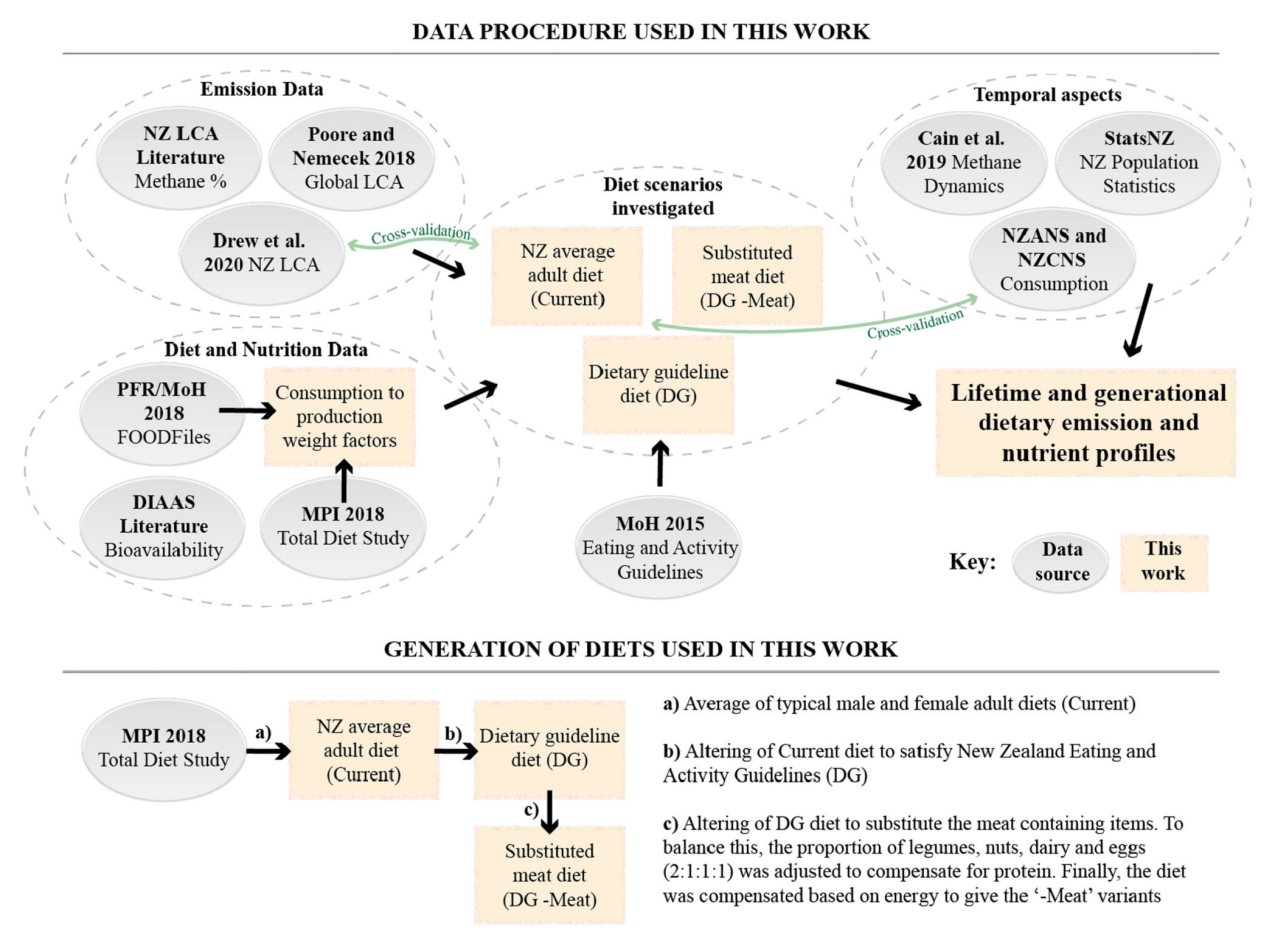
A diagrammatic representation of the interacting methodologies and data sources used in this work.

As this work involves several interacting data sources and methodological approaches, a diagrammatic representation is also included (Figure 1). This details the three general domains of data: 1) diet and nutrition data, 2) emissions data, and 3) temporal data, and the two methodological domains involving: 1) basic emission and nutrient information for dietary scenarios and 2) lifetime and generational dietary emission profiles.

## 3 Results

**Figure 2:**
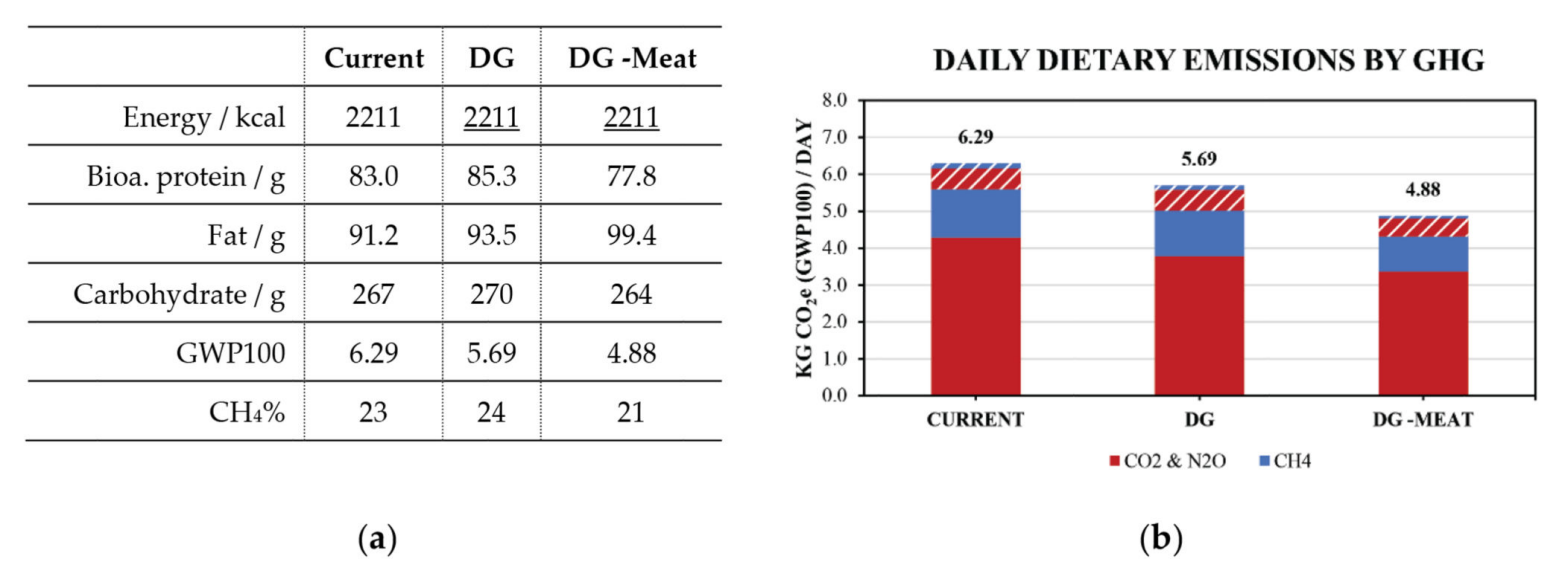
Estimated daily dietary emissions for the reference ‘Current’ diet and the hypothetical ‘DG’, ‘DG -Meat’ by energy compensation. Emissions are allocated by emissions due to consumption (solid areas) and emissions due to waste (dashed areas) while the colours differentiate emissions of CH_4_ (blue) from emissions of CO_2_ and N_2_O (red).

A summary of daily values for the diets explored here is provided in Table 1 and a visual representation is presented in Figure 2. The estimated dietary emissions from the Current diet is within 5% of the estimate from Drew, Cleghorn, Macmillan and Mizdrak [13], indicating good consistency between the studies, even though different diet frameworks were used. The two hypothetical diets result in altered daily emissions between 0.61 and 1.42 kg CO_2e_ per day from the Current baseline. The DG -Meat hypothetical diet, where meat is substituted while retaining energy intake, resulted in the lowest daily dietary emission value (4.88 versus 6.29 kg CO2e per day), consistent with literature reporting the positive relationship between meat and dietary emissions [13–15]. These changes were associated with changes in both short- and long-lived gases, often leading to small a reduction in the proportion of CH_4_ in daily emission values (e.g., 23% versus 21% for Current and DG-Meat).

**Figure 3:**
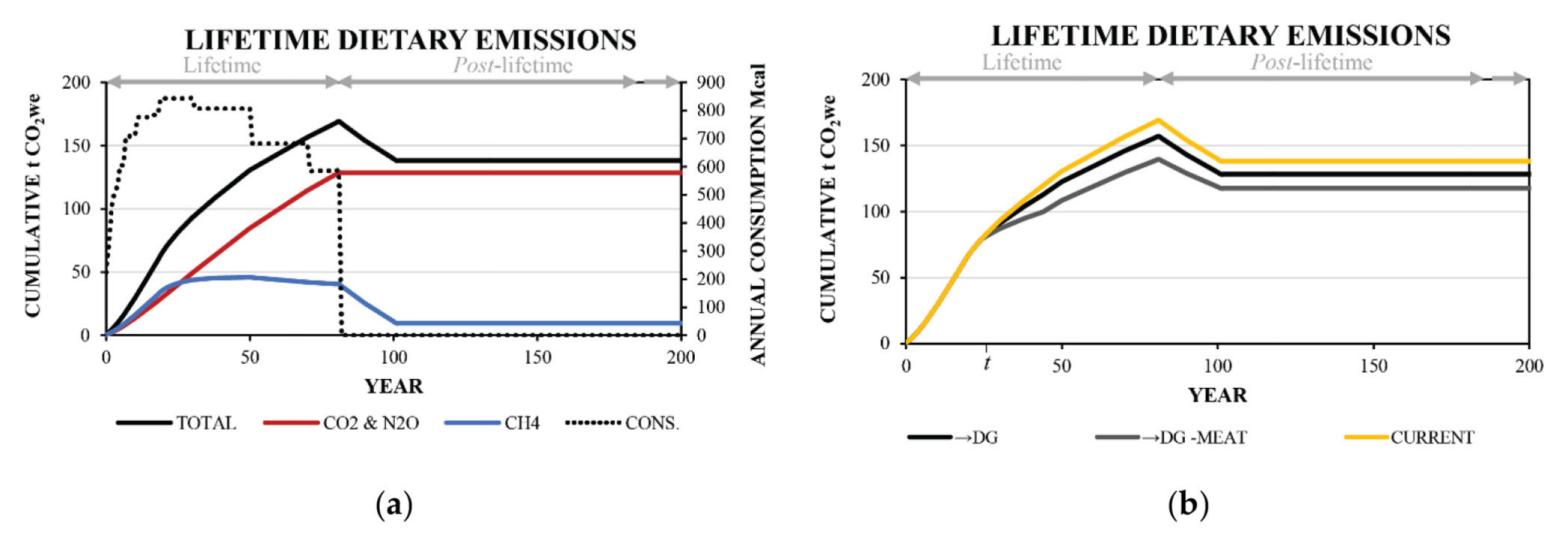
a) Cumulative lifetime dietary emission profiles for the Current diet showing the differentiation of CH_4_ and CO_2_ and N_2_O, and b) the cumulative lifetime dietary emission profiles using GWP* for the →DG and →DG -Meat diet transitions from the Current reference diet. The annual energy consumption at a given individual life stage is given by the dotted line in a). t denotes the 25th year where a transition from the Current diet to the hypothetical diets occurs.

By applying the transition lifetime modelling scenario, the short- and long-term behaviours of CH_4_ are explored (Figure 3a). Over the first twenty years of the lifetime, the strong contribution from GWP* flow term results in a large contribution from CH_4_ to the total cumulative emission CO_2we_ values (in conjunction with the strong short-term warming impact of CH_4_ emissions). After this time, the contribution from the stock-term decreases, particularly after the 19 to 30-year life stage, when the annual energy consumption and the annual emission of CH_4_ begin to decrease. Throughout the modelled lifetime, the CO_2_ and N_2_O emission proportions are directly related to the annual energy consumption and the cumulative emission value for these gases undergoes a close to linear increase from birth to death.

In Figure 3b, dietary transitions (denoted by →) from the Current diet to the alternative hypothetical diets are explored alongside the reference Current diet. The Current diet involves a total of 131 and 140 cumulative tonnes of CO_2we_ in the 50^th^ and 100^th^ years, respectively. For the transitions, divergence in the cumulative emission CO_2we_ values is observed immediately after the 25^th^ year. For the →DG diet transition this divergence involves a total of 123 and 130 cumulative tonnes of CO_2we_ in the 50^th^ and 100^th^ years, respectively; a reduction of 6 and 7% compared with the Current diet, respectively. By comparison, for the →DG -Meat diet transition, this results in 108 and 119 cumulative tonnes of CO_2we_ in the 50^th^ and 100^th^ years, respectively; a reduction of 17 and 15% compared with the Current diet, respectively, or 12 and 8% relative to the →DG diet. The variation in cumulative warming between scenarios slowly decreases over time and, on death of the modelled individual, there is a rapid reduction in the absolute difference and a small reduction in the relative difference between all diet scenarios (Table 2). This reduction in cumulative warming from CH_4_ arises due to the cessation of emissions and the reversing of sign for the GWP* flow term. Twenty years after death (and the last annual emission), the cumulative impact reaches a plateau or a long-term limit that equals the cumulative total GWP100 values for CO_2_, N_2_O and 0.25x the cumulative total GWP100 value for CH_4_.

When comparing the results from the daily GWP100 values to the lifetime modelling using both GWP100 and GWP*, two considerations arise (Table 2 and Figure A3a). Firstly, the relative difference between stand-alone diets is larger for the daily GWP100 values than the transition cumulative impacts (both GWP100 and GWP*), which arises primarily due to the transition where a portion of the lifecycle is spent consuming the Current diet. Secondly, the lifetime cumulative impact is underestimated at year 50 (-14%) and overestimated at year 100 (+19%) by GWP100 when compared with GWP*, as the flow dynamics of CH_4_ on atmospheric warming are not clearly accounted for. Specifically, because it is a short-lived gas, the warming caused by CH_4_ is primarily determined by recent emissions, in contrast to the long-term cumulative effects from CO_2_ and N_2_O. Despite these relative changes, the order of climate impact remains the same: Current diet > →DG diet transition > →DG -Meat diet transition.

While this analysis and similar analyses in the literature, such as Drew, Cleghorn, Macmillan and Mizdrak [13], have a strong focus on estimating dietary emissions, it is useful to acknowledge that nutrient profiles associated with the proposed hypothetical diets are important in the context of sustainability. To help these considerations, the nutrient profiles and adult nutrient reference values (NRVs) for the reference Current diet and the hypothetical DG and DG -Meat transition diets are included in Table ESI4 to illustrate how these diets compare with recommended nutrient intakes. For the Current diet, estimated intakes were below recommendations for calcium (-21%), dietary folate (-11%), polyunsaturated fatty acids (-50%), dietary fibre (-10%), magnesium (-5%), and pantothenic acid (-25%), and in excess for total fat (+27%), saturated fatty acids (36%),. and sodium (34%). For the hypothetical DG diet, improvements are made for calcium, dietary folate, dietary fibre, magnesium and sodium; furthermore, none of the inadequate intakes identified in the New Zealand Adult Nutrition Survey (calcium, selenium, vitamin A, vitamin B6 and zinc) [51] are apparent for the DG diet. For the hypothetical DG -Meat diet, the meeting of NRVs are generally like the baseline DG diet, although some characteristic differences are observed. For example, the recommended daily intake for iron was not met (-10%) because of the removal of the iron associated with red meat consumption. There is also a trade-off between sugars and protein when compared with the DG diet using an energy compensation of 101 g total sugars per day and 78 g protein per day versus 95 g total sugars per day and 85 g bioavailable protein per day (i.e., 6% greater sugar intake for a 9% reduction in the intake of bioavailable protein in the DG diet). In summary, the substitution of meat containing items from these hypothetical diets is not achieved without impact on the nutrient composition, as has been reported in the literature for actual diets [16]. Although these changes in nutrient composition can be mitigated through diet optimisation or through supplementation/fortification, for the average individual, these changes need to be considered against their effects on the personal emission profile. Future studies should consider the bioavailability of nutrients or information-limited nutrients (such as essential amino acids) and the nutrient cost of dietary patterns/carbon costs per nutrient [53–55]. Overall, sustainability studies must shift from an energy-sufficient diet approach to one that considers a nutrient-adequate diet, and this aspect is explored in a sensitivity analysis below.

### 3.1 Sensitvity analysis

To test and explore the assumptions and data associated with dietary modelling, several additional analyses were undertaken. These include compensating diets using protein, different proportions of short- and long-lived GHGs and considering how the results compare if the modelling is extended from an individual perspective to a collective or family perspective. To infer that, New Zealand can be an outlier in the global context due to relatively high production efficiencies (GHG/kg food produced), an additional analysis using median global LCA data is also carried out. However, due to limitations for comparisons between global and New Zealand LCA data, this has been included in the appendix for reference purposes only.

#### 3.1.1 Compensation based on nutrients: protein

**Figure 4:**
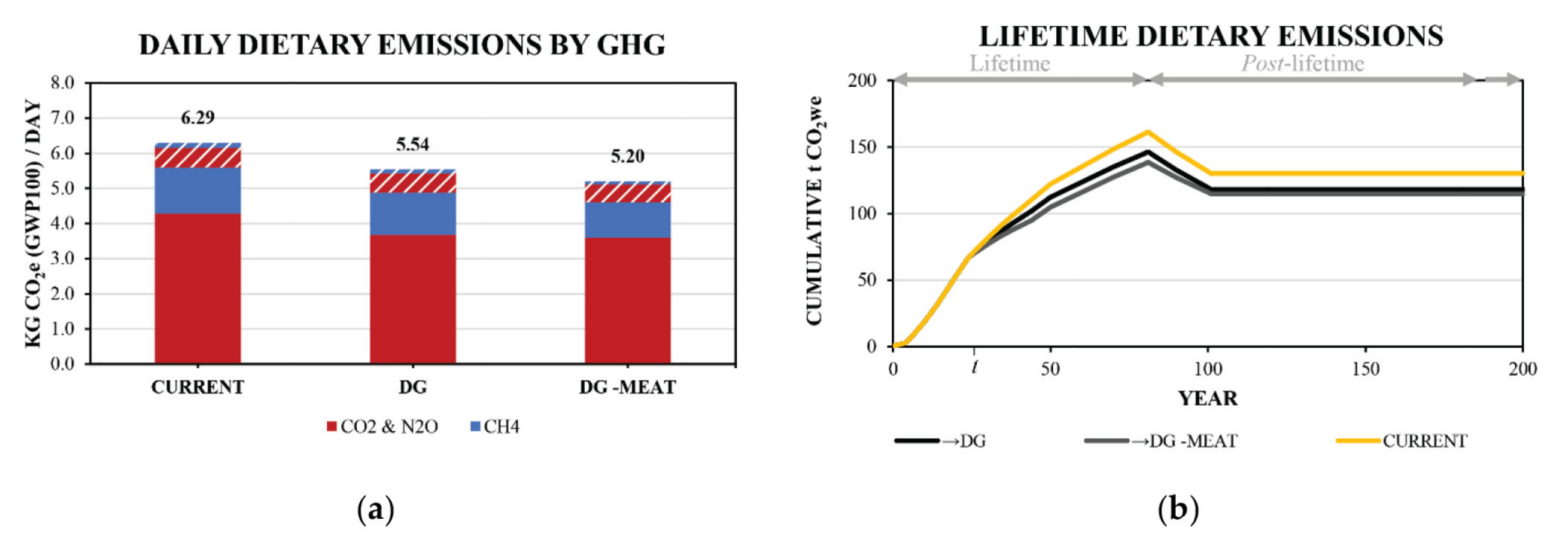
a) Daily estimated dietary emissions including emissions due to waste in dashed areas and b) cumulative lifetime transition dietary emissions using GWP* and protein compensation rather than energy compensation used in Figure 2 and 3b, respectively. t denotes the 25^th^ year where a transition from the Current diet to the hypothetical diets occurs.

It is common practice in related literature to compensate diets based on their energy prior to comparison [12,14,18], as in the above section. An alternative compensation approach to the energy approach reported earlier would be to consider protein (including protein quality, amino acid composition or bioavailability). Recent work by Loveday [56] using LCA data from the [15] database, identified that the protein quality of food items can alter the qualitative estimation of environmental impacts. Given that on a per serving, unprocessed animal-source foods are often much higher in protein content and protein bioavailability [10,53–55,57] and can be lower in calories per g of bioavailable protein than some plant-source foods, choosing either energy or protein compensations has the potential to provide different outcomes. Moreover, protein-rich animal-source foods (including milk and milk products) have been reported to be the lowest-cost source of several essential micronutrients (in bioavailable form) in international literature [53–55,57,58]. This approach is used to compare the hypothetical →DG and →DG -Meat diet transitions with the reference Current diet (Figure 4a) and propagated based on protein consumption throughout the lifetime (Figure 4b).

With protein compensation, the Current diet involves a total of 122 and 132 cumulative tonnes of CO_2we_ in the 50^th^ and 100^th^ years, respectively. The →DG diet transition involves a total of 112 and 119 cumulative tonnes of CO_2we_ in the 50^th^ and 100^th^ years, respectively: a reduction of 8 and 9% compared with the Current diet, respectively. By comparison, for the →DG -Meat diet transition this involves 105 and 116 cumulative tonnes of CO_2we_ in the 50^th^ and 100^th^ years, respectively: a reduction of 14 and 12% compared with the Current diet, respectively, and 6 and 3% relative to the →DG diet transition, respectively. The changing of relative differences for the two transition scenarios arises due to an increased (→DG) or decreased (→DG -Meat) bioavailable protein density per kg CO_2we_ for the diet when compared with the current diet. Despite these differences, the order of climate impact remains the same when using energy and protein compensation: Current diet > →DG diet transition > →DG -Meat diet transition; but, the effect of transitioning to the meat-free diet is more than 50% smaller than when considered on an energy basis.

It is notable that when compensating by protein, the Current and DG and DG -Meat diet amount to total energy values of 2211, 2152 and 2360 Kcal per day, respectively, to achieve an equivalent bioavailable protein value of 83 g per day. The above analysis highlights that there is a potential impact of using energy compensation on both the overall climate impact, but also for the oversupply of energy in the DG -meat diet relative the Current or DG diets, where it is important to maintain a constant protein consumption.

#### 3.1.2 The potential for long-term warming legacies: different meat choices

**Figure 5:**
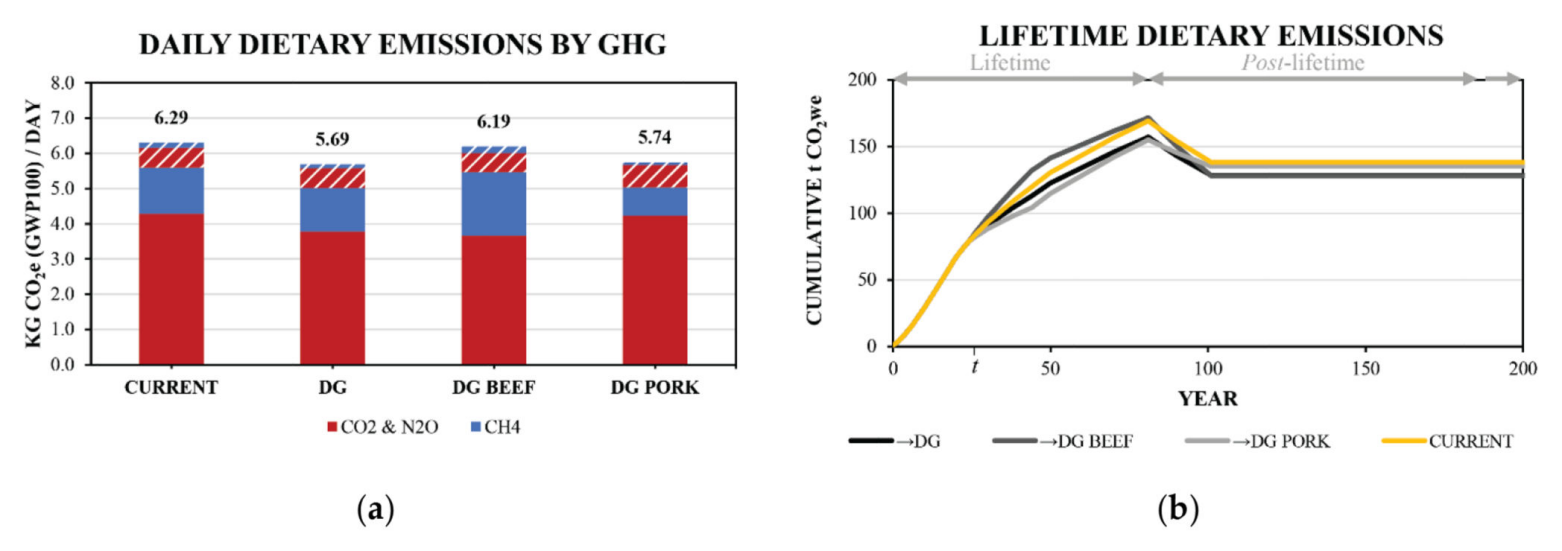
a) Daily estimated dietary emissions including emissions due to waste in dashed and b) lifetime dietary emissions for the baseline average adult diet and hypothetical diets where all core meat is allocated to either beef or pork items using a protein compensation. t denotes the 25^th^ year where a transition from the Current diet to the hypothetical diets occurs.

Given that the production of different meat items can emit different amounts and proportions of short- and long-lived GHGs, meat choice can serve as a good case study for contrasting the warming impacts of diets with significantly different proportions of CH_4_. In addition, it is likely that the allocation of meat items in an individual’s actual diet may vary considerably from those in the baseline average adult diet. This may be exacerbated by individual preferences, including those formed in accordance with recommendations made in literature, which encourage or discourage particular types of meat (e.g., white vs. red meats) [14]. For this analysis, the total proportion of non-discretionary meat-based foods of the base DG diet were allocated solely to one meat category (e.g., beef or pork) and results are detailed in Figure 5 and in Table 4.

With meat allocation to either beef or pork, the cumulative impact differs from the baseline →DG diet transition. An interesting observation is that, in the short-term, the allocation to pork reduces the cumulative impact (6% at year 50), while in the long-term, the cumulative impact is increased for this diet (5% at year 100) compared with the baseline. In contrast, transition to beef has an increased short-term cumulative impact (15% at year 50), but no effect over the 100-year term (<1% at year 100) compared with the baseline. In other words, a transition to pork has a lower impact over an individual’s lifetime compared with a transition to beef; however, the long-term impact of the transition to beef is removed soon after consumption stops, while much of the long-term impact of a transition to pork remains indefinitely. Importantly, this behaviour is not reflected by the daily GWP100 values of DG Beef and DG Pork diets of 6.19 and 5.75 Kg CO2e per day, respectively, which would imply a reduced impact for the →DG Pork diet transition over the →DG Beef diet transition. These hypothetical diet transitions are good examples of the different climate impacts of CH_4_ and CO_2_, where the higher GWP100 value and CH_4_ proportion for beef is offset over time by the primarily CO_2_ and N_2_O emissions associated with pork.

The key interpretation from this analysis is that care should be taken when comparing items that emit considerably different proportions of short- and long-term GHGs, such as CH_4_ and CO_2_, respectively. Failing to do this can risk causing unintended perverse outcomes, such as increased cumulative warming impact, particularly in the longer-term. To alleviate the uncertainty associated with the overall GWP100 values indicated above, an estimation of the long-term impact of CH_4_ from an individual’s food can be appreciated using the stock GWP* term (0.25 × GWP100_CH_4__). By combining this stock term contribution and the total CO_2_ and N_2_O GWP100 values, a long-term index to compare foods on an estimated long-term impact can be allocated. This index correlated with the GWP100 values can differentiate the short- and long-term impacts of different foods (Figure A8).

#### 3.1.3 Considering a collective perspective: the role of descendants

**Figure 6:**
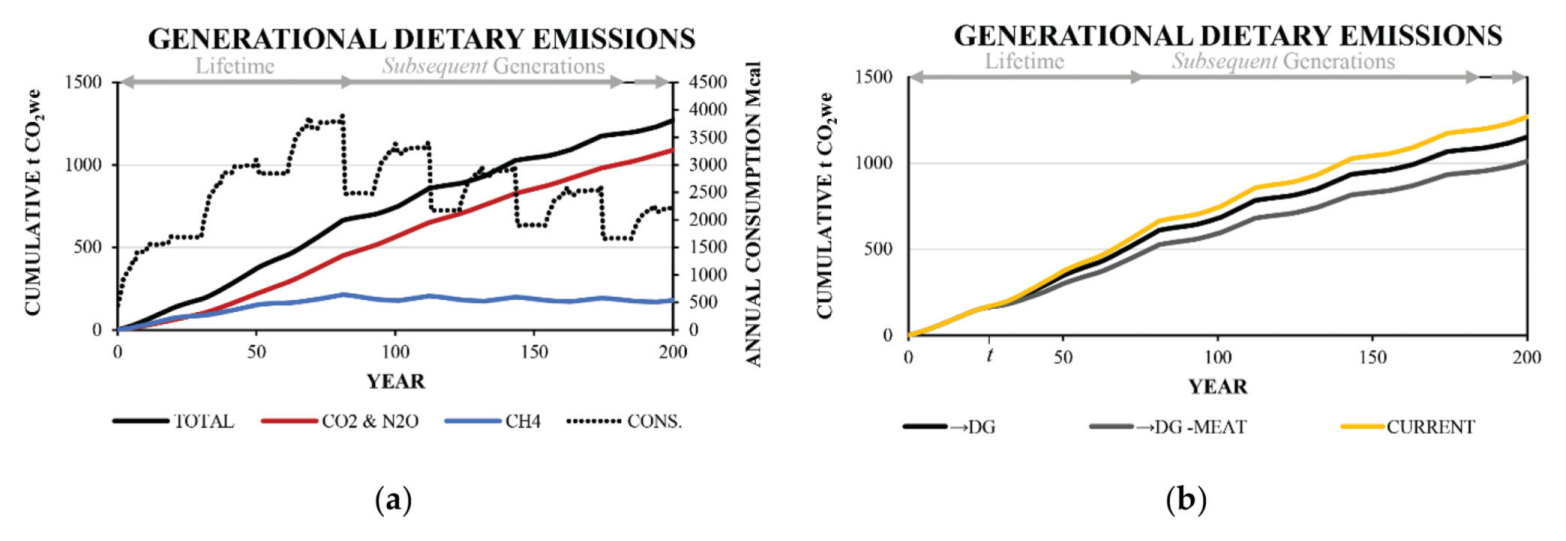
a) Cumulative generational dietary emission profiles for the Current diet showing the differentiation of CH_4_ and CO_2_ and N_2_O, and b) the cumulative generational dietary emission profiles for the oDG and oDG -Meat diet transitions. The energy consumption each year is shown by the dotted line in a). t denotes the 25^th^ year where a transition from the Current diet to the hypothetical diets occurs.

An important consideration of the individual perspective explored above (i.e., a singular lifetime) is that the dynamics and interpretations are not the same for a collective or family perspective. As an intermediate step towards a broader perspective, a generational dietary emission impact can also be considered. In this case, the responsibility of descendants is allocated to the direct family line (i.e., and considers the dietary impact of descendant lines consumption (Figure A3). The cumulative dietary emissions of the reference Current and hypothetical →DG and →DG -Meat diet transitions are shown in Figure 6b and Table 5.

As in the individual transition case above, the CH_4_ contribution to the overall cumulative emission CO_2we_ values increases until reaching the peak energy consumption. In this case, peak consumption occurs on death of the modelled 0^th^ generation in the 82^nd^ year, after which time the consumption decrease due to the less than replacement-level New Zealand total fertility rate. After this time point, the contribution from CH_4_ is progressively decreasing towards the long-term CH_4_ limit, while the contribution from CO_2_ and N_2_O increases (Figure 6a). The generational modelling differs from modelling in the prior section as it assumes an ongoing emission scenario and no rapid reduction towards a dominance of the static stock term at 0.25x the GWP100 for the CH_4_ proportion occurs, although a slow divergence over time does occur (Figure A3b). The ongoing nature of the emission series means that there is better consistency between the cumulative emissions calculated with GWP100 compared with using GWP* metric for CH_4_ (Figure A5b). In addition, the relative reductions due to dietary changes are well approximated by the relative differences between respective daily GWP100 values.

To explore the influence of the total fertility rate on cumulative dietary emissions under the generational scenario, a hypothetical total fertility rate of 2.25 was also used (Figure A6a). This value was chosen based on the vicinity of the actual total fertility rate of 1.75 to a replacement level, and as it is well within documented historic fertility rates for New Zealand [59]. In this case, an increased fertility rate has a strong impact on the cumulative total in the long term. For example, the average changes in cumulative emission CO_2we_ values for the year 100 and 200 are 28% and 93% higher, respectively, when the 2.25 total fertility rate is used compared with a total fertility rate of 1.75.

A key insight that GWP* facilitates is the acknowledgement that intertemporal patterns of consumption can imply different results. For example, populations that are growing quickly (i.e., a total fertility rate above the replacement level or net immigration) would experience substantial warming from CH_4_ emitting items within a diet, even if the diet itself remained the same over time. Furthermore, a population of fixed size that increases its meat intake substantially will also experience warming over the period across which that increase takes place. GWP* will reveal the warming dynamics resulting from any given emission pathway, but interpreting what this implies regarding, for example, equitable mitigation efforts or overall ‘sustainability’ required additional considerations noting these contextual issues.

## 4 Discussion

### 4.1 Acknowledging dietary emissions in context

When considering dietary emissions at an individual level it is useful to ground these in the broader individual emission context. In New Zealand the estimated proportion of household consumption-based emissions for ‘food and non-alcoholic beverages’ is around 26%^1^ averaged for the years 2011 to 2017 [60]. Using this estimate and assuming that household emissions have negligible short-term GHG contributions from other sectors (i.e., CH_4_), an individual’s deliberate dietary shift to a the hypothetical DG or DG -Meat diets may decrease an individual’s consumption-based total warming footprint by 2 to 4% (depending on energy or protein compensation) at year 100. For context, the transition to the hypothetical DG or DG -Meat diet would equate to a reduction in ‘transport’ or ‘housing and household utility’ consumption-based emissions of around 5 to 11% or 14 to 29%, respectively [60]. Another way to contextualise the difference in cumulative warming is to compare the transitions with the carbon footprint of international flights. For example, the transition from the Current diet to the DG diet (-10 t CO_2we_) in at age 25 would equate to avoiding around three return international flights (3.5 t CO_2_ per return) from Auckland, New Zealand to London Heathrow, United Kingdom [61] over a lifetime; transitioning from the Current diet to the DG -Meat diet (-21 t CO_2we_) would equate to avoiding around six. As the emissions context is highly dependent on the individual, it remains unclear how actual reductions in dietary emissions may compare with potential mitigation options in the other emission areas.

Understanding the climate change impacts of food production is an important part of wholistic sustainability approaches; however, the myriad of other environmental issues is also relevant. For example, LCA reviews have highlighted the various environmental impact categories that have been used to compare across different food items and food groups [28,29]. These reviews found that GHG emissions were primarily of interest in literature (>90% of studies), while other impacts such as land use, water use, eutrophication and acidification are infrequently used (<30% of studies in each case). A singular focus of sustainability endeavours on dietary emissions should be avoided, as this creates a risk of ‘burden-shifting’ or improving one stage in the supply chain or impact of concern, by shifting the problem to another stage in the chain or onto a different concern.

### 4.2 Clarity over the dynamics of GHGs

The GWP* results highlight interesting dynamics, such as the cumulative warming impacts of the dietary CH_4_ emissions decreasing to near zero after the death of the individual. This results in a smaller absolute difference between the hypothetical diet transitions and Current diet at 100 years when compared with a daily GWP100 value or the cumulative GWP100 values. In simple terms, CO_2_ and N_2_O emissions accumulate over the long-term to become the dominant factor of the cumulative emission CO_2we_ values. In the long run, an individual’s warming contributions are likely to be dominated by their CO_2_ emissions and dietary transitions evaluated in this study may allow a reduction of 2 to 4% to this total, based on New Zealand consumption-based emission patterns [60]. However, it should also be noted that CH_4_ emissions can constitute a major component of the cumulative emission CO_2we_ values in the short-term, particularly from year 0 to around year 45. Both points relate to the ongoing discussion of how best to prioritise mitigation of short-, medium- and long-term climate impacts [62].

An important distinction should be made between this study and the recent application of GWP* to dietary emissions by Ridoutt, Baird and Hendrie [36]. The Ridoutt, Baird and Hendrie [36] study uses GWP* to provide estimates of relative dietary warming given trends in production between 1990 and 2018 (i.e., setting 1990 as the reference for temperature contribution). This approach assumes that trends between these years are ongoing, or in other words, sectors that are reducing CH_4_ emissions per unit of product will continue to reduce CH_4_ by this proportion. Due to the dominant impact of the flow term of GWP*, deviations from these assumed trends will have a strong impact on the estimate of dietary emissions. By contrast, this work used GWP* to explore the cumulative warming profile of an individual’s lifetime (i.e., setting zero emissions as a reference). This approach captures the strong short-term warming, when atmospheric

concentrations of CH_4_ increase, neutral warming impacts as emission intensities are roughly stable, and the decrease in warming once emission intensity decreases or stops. These two unique applications highlight how GWP* is flexible in how it can be applied and that it can be used to provide different information (i.e., relative and marginal warming).

### 4.3 Limitations and considerations

The key limitation of the modelling undertaken, is the application of one or two dietary composition throughout different life stages using consumption profiles, given that actual diet compositions change over time. While this approach provides a satisfactory approximation of dietary emission over various life stages (Figure A2), more detailed analyses are recommended to understand how the trajectory of nutrient adequacy over the course of life stages. Furthermore, the underlying datasets used in these simulations carry with them important considerations and limitations.

The New Zealand Adult and Child Nutrition Surveys [50,51], which underpin the simulated Total Diet Survey framework used here, were undertaken in 2008/2009 and 2002, respectively. These surveys represent the latest national surveys for the respective age groups and highlight the lack of more up-to-date diet composition data for the broader New Zealand population. Based on national indicators [63,64] and international research [65,66], it is expected that actual typical New Zealand diets have changed since these surveys were undertaken.

LCA data both globally and specific to New Zealand are limited. While works by Poore and Nemecek [15] and Drew, Cleghorn, Macmillan and Mizdrak [13] represent two of the most robust LCA databases, they both have a heavy reliance on LCA literature published between 2000 and 2010. It is acknowledged that LCA methods do have some limitations [31,67] and do not consider all aspects of an impact category. For example, soil sequestration [68] and on-farm sequestration [69] of carbon are rarely accounted for and this is expected to vary by land use (e.g., pasture systems versus cropping systems). Having an opposing impact, the potential to use spared agricultural land for carbon sequestration has been highlighted as an additional climate benefit of changing diets, beyond the direct emission reductions [70]. However, such estimates of potential land repurposing should take care to consider the suitability of land for other uses and the impacts of the repurposing process [4]. These more complex land-use related impacts were outside the scope of this study, where we explored only direct dietary emissions as available from the best available database on food emissions.

Despite efforts to standardise [31,67,71], contrasting LCA methodologies remain a challenge and can be exemplified in the case of different IPCC GWP100 equivalence values and LCA stages captured by these databases (although efforts were made to standardise these aspects by both databases). Particular considerations should include: 1) the global dataset was harmonised and with recalculations of emissions due to land-use change and above-ground sequestration; in comparison, the New Zealand dataset was not, 2) the global dataset had less representation of processed products which hindered data matching compared with the New Zealand dataset and 3) the New Zealand dataset had some consideration of the transport required to deliver the products to New Zealand retail outlets while the global dataset does not. It is also noted that emissions resulting from post-retail activities other than waste were not accounted for (such as the emissions due to cooking or preparation).

The proportion of food wasted is poorly quantified. Due to the lack of New Zealand-specific waste data at a product-level the UK Waste and Resources Action Programme data [48] were used. While these data are arguably the most appropriate available, they are limited with respect to the general product types that they cover. These factors highlight that the proportion of waste emissions is expected to have a large degree of uncertainty.

It is acknowledged that various aspects included in this modelling are expected to show temporal trends. For example, agricultural efficiency increases larger than 1% per year were observed globally for the time period between 1990 to 2015 [72], therefore the emissions per kilogram of product would be expected to generally decrease over time. Variables relating to LCA data, climate impacts for different GHGs, typical diets, food nutrient profiles and waste, amongst others, are expected to have both temporal variation and temporal trends. Without robust datasets to base trends for these variables, no corrections for such trends have been attempted and these aspects are considered outside of the scope of this investigation. Noting the strong interaction of trends with warming estimated by GWP* there is a need for better understanding of these aspects.

## 5 Conclusions

There is significant interest for using dietary transitions to improve the nutrient and climate outcomes of the food system. Care is needed to ensure replacement diets are indeed nutritious and that climate benefits are adequately accounted for. Diet-related GHG emissions are an important part of an individual’s carbon footprint and, as many recent works have highlighted, they can be lowered by eliminating meat in the diet. However, the stock and flow behaviour of how dietary emissions contribute to warming over time is not always clear in these studies (i.e., by the comparison of CO_2e_ values). In this study, dietary guidelines are used to improve the average diet (i.e., ‘DG’) and the climate impact of substituting meat (i.e., ‘DG -Meat) is explored for a consumer of New Zealand-produced meat. A transition at age 25 to the hypothetical DG diet reduces the cumulative warming associated with food consumption by 7 to 9% at the 100^th^ year compared with consuming the average New Zealand diet. The reduction in cumulative warming associated with food consumption from the transition to a hypothetical meat-substituted diet varied between 12 and 15%. Using New Zealand consumption-based emission estimates as context, transitions to these hypothetical diets may reduce an average individual’s lifetime warming contribution from consumption-based activities by 2 to 4%. In addition to GWP*, the *de facto* GWP100 metric was also used and, regardless of methodology, the order of climate impact for the scenarios explored remained the same.

While CH_4_ is responsible for a large part of an individual’s cumulative CO_2we_ dietary emissions, the long-term impacts of CO_2_ and N_2_O should not be neglected as a result of a focus on CH_4_, as these end up having a greater warming effect across a whole lifetime, and are not rapidly reversed once consumption ceases, unlike for CH_4_. GWP* provides a useful tool to analyse the impact of different foods on a person’s consumption footprint and highlights the strong warming of CH_4_ when emissions are increasing, but also the stabilisation or reduction in warming when emissions are approximately stable or decreasing. This contrasts with the behaviour of longer-lived GHGs, such as CO_2_ and N_2_O, whose warming effect accumulates over time. Moreover, these different physical behaviours of GHGs have important trend-specific implications for which (and how) different GHGs are prioritised for reduction or offsetting, as well as for improving the understanding of the long-term climate sustainability of the food system in the broader context of national and global emission accounting.

Importantly, there are general improvements in nutrient intake by adopting the dietary guidelines compared with the average New Zealand diet. However, the DG and DG-Meat diets are not nutrient equivalent as substituting meat results in characteristic nutrient differences. This work emphasises the need to ensure dietary transitions are sustainable from both nutritional and climate perspectives.

Further work in this area should seek to understand nutrient considerations more-completely (particularly for information-limited nutrients, such as essential amino acids), account for developing areas related to the climate change impact category (particularly related to land-use) and consider a broader range of sustainability indicators (such as affordability). In addition, there is an opportunity to explore how individual actions and impacts estimated with GWP* compare and contrast with collective-level outcomes where factors such as population dynamics become important.

## Supplementary Material

Supplementary Material

## Figures and Tables

**Table 1:** Summary of the nutrient and emission daily values for Current, DG and DG -Meat diets used in this work. Energy compensation (the nutrient kept constant with respect to the Current diet) is used for DG and DG -Meat diets. Protein values include bioavailability. Additional nutrient and emission data is given in Tables ESI3 to ESI5.

	Current	DG	DG -Meat
Energy / kcal	2211	2211	2211
Bioa. protein / g	83.0	85.3	77.8
Fat / g	91.2	93.5	99.4
Carbohydrate / g	267	270	264
GWP100	6.29	5.69	4.88
CH_4_%	23	24	21

**Table 2:** Emission data for the different diet scenarios for the daily GWP100, lifetime GWP100 and lifetime GWP* values using energy compensation. * denotes the comparison (Δ%) between the GWP100 and GWP* values.

				Lifetime GWP100				Lifetime GWP*				Δ%*	Δ%*
	Daily GWP100			Cumulative t CO_2e_				Cumulative t CO_2we_				Year	Year
Diet	Kg CO_2e_/day	CH_4_%	Δ%	Year 50	Δ%	Year 100	Δ%*	Year 50	Δ%	Year 100	Δ%	50	100
→DG	5.69	24%	-10%	104	-3%	156	-7%	123	-6%	130	-7%	-14%	20%
→DG -Meat	4.88	21%	-23%	96	-11%	141	-16%	108	-17%	119	-15%	-10%	18%
Current	6.29	23%	-	108	-	167	-	131	-	140	-	-16%	20%

**Table 3:** Emission data for the different diet scenarios for the daily GWP100, lifetime GWP100 and lifetime GWP* values using energy compensation. * denotes the comparison (Δ%) between the GWP100 and GWP* values.

				Lifetime GWP100				Lifetime GWP*				Δ%*	Δ%*
	Daily GWP100			Cumulative t CO_2e_				Cumulative t CO_2we_				Year	Year
Diet	Kg CO_2e_/day	CH_4_%	Δ%	Year 50	Δ%	Year 100	Δ%*	Year 50	Δ%	Year 100	Δ%	50	100
→DG	5.54	24%	-12%	93	-5%	143	-9%	112	-8%	119	-9%	-16%	20%
→DG -Meat	5.20	21%	-17%	90	-8%	137	-13%	105	-14%	116	-12%	-13%	18%
Current	6.30	23%	-	98	-	157	-	122	-	132	-	-19%	20%

**Table 4:** Emission data for the different diet scenarios for the daily GWP100, lifetime GWP100 and lifetime GWP* values using energy compensation. * denotes the comparison (Δ%) between the GWP100 and GWP* values.

				Lifetime GWP100				Lifetime GWP*				Δ%*
	Daily GWP100			Cumulative t CO_2e_				Cumulative t CO_2we_				Year
Diet	Kg CO_2e_/day	CH_4_%	Δ%	Year 50	Δ%	Year 100	Δ%*	Year 50	Δ%	Year 100	Δ%	100
→DG	5.69	24%	-	104	-	156	-	123	-	130	-	20%
→DG Beef	6.19	33%	9%	109	5%	165	6%	141	15%	130	0%	27%
→DG Pork	5.75	16%	1%	105	1%	157	1%	115	-6%	136	5%	15%
Current	6.29	23%	11%	108	4%	167	7%	131	7%	140	8%	20%

**Table 5:** Emission data for the different diet scenarios for the generational GWP* values using a total fertility rate of 1.75 and 2.25. * denotes the comparison (Δ%) between the TFR values of 1.75 and 2.25.

				Lifetime GWP100				Lifetime GWP*				Δ%*	Δ%*
	Daily GWP100			Cumulative t CO_2e_				Cumulative t CO_2we_				Year	Year
Diet	Kg CO_2e_/day	CH_4_%	Δ%	Year 50	Δ%	Year 100	Δ%*	Year 50	Δ%	Year 100	Δ%	100	200
→DG	5.69	24%	-10%	680	-8%	1152	-9%	876	9%	2237	-9%	29%	94%
→DG -Meat	4.88	21%	-23%	592	-20%	1010	-20%	756	-21%	1937	-22%	28%	92%
Current	6.29	23%	-	743	-	1269	-	958	-	2468	-	29%	94%

## Data Availability

Supporting data used in this work is provided in the electronic supplementary materials and additional material is provided in the appendix.

## Footnotes

1 The other key emission areas averaged over the same period are: ‘transport’ at 35%, ‘housing and household utilities’ at 14% and ‘recreation and culture’ at 5%.
