# Supplementary material for: Lifetime climate impacts of diet transitions: a novel climate change accounting perspective
Source: Sustainability. Author manuscript; Available in PMC 2021 Jun 22. (PMC7611040; doi:10.3390/su13105568)
Supplement: Supplementary Material [file EMS126081-supplement-Supplementary_Material.docx]

**Supplementary Materials and Appendix:** The following are available online at www.mdpi.com/xxx/s1, Table ESI1: Matching table used to link various data sources, Table ESI2: Composite food construction for Global LCA data, Table ESI3: Consumed amount per day for each diet; servings per food group, Table ESI4: Nutrition data for each diet by energy and protein compensation, Table ESI5: NZ and Global emission data for each diet by energy and protein compensation, Table ESI6: NZ LCA data from Drew et al. 2020 with modifications, Table ESI7: Global median LCA data from Poore and Nemecek 2018 with modifications, Table ESI8: UK-based waste data, Table ESI9: DIAAS protein conversion factors for different food groups, Table ESI10: Nutrient reference values. A separate appendix file has been provided and details additional material.
